# Engineering a reagentless biosensor for single-stranded DNA to measure real-time helicase activity in *Bacillus*

**DOI:** 10.1016/j.bios.2014.06.011

**Published:** 2014-11-15

**Authors:** Matthew Green, Neville S. Gilhooly, Shahriar Abedeen, David J. Scott, Mark S. Dillingham, Panos Soultanas

**Affiliations:** a School of Chemistry, Centre for Biomolecular Sciences, University of Nottingham, University Park, Nottingham NG7 2RD, UK; b School of Biosciences, University of Nottingham, Sutton Bonington, Leicestershire LE12 5RD, UK; c School of Biochemistry, Medical Sciences Building, University of Bristol, Bristol BS8 1TD, UK

**Keywords:** DNA, (deoxynucleic acid), SSB, (single strand DNA binding protein), ssDNA, (single stranded DNA), IDCC, N-[2-(iodoacetamido)ethyl]-7-diethylaminocoumarin3-carboxamide, MDCC, 7-diethylamino-3-((((2-maleimidyl)ethyl)amino)carbonyl) coumarin, FDA5M, fluorescein diacetate 5 maleimide, EMSA, electrophoretic mobility shift assay, DTT, dithiothreitol, EDTA, ethelenediaminetetraacetic acid, TBE, Tris boric acid ethelenediaminetetraacetic acid, SSB, Helicases, Fluorescence, DNA unwinding, *Bacillus subtilis*, Firmicutes

## Abstract

Single-stranded DNA-binding protein (SSB) is a well characterized ubiquitous and essential bacterial protein involved in almost all aspects of DNA metabolism. Using the *Bacillus subtilis* SSB we have generated a reagentless SSB biosensor that can be used as a helicase probe in *B. subtilis* and closely related gram positive bacteria. We have demonstrated the utility of the probe in a DNA unwinding reaction using a helicase from *Bacillus* and for the first time, characterized the *B. subtilis* SSB's DNA binding mode switching and stoichiometry. The importance of SSB in DNA metabolism is not limited to simply binding and protecting ssDNA during DNA replication, as previously thought. It interacts with an array of partner proteins to coordinate many different aspects of DNA metabolism. In most cases its interactions with partner proteins is species-specific and for this reason, knowing how to produce and use cognate reagentless SSB biosensors in different bacteria is critical. Here we explain how to produce a *B. subtilis* SSB probe that exhibits 9-fold fluorescence increase upon binding to single stranded DNA and can be used in all related gram positive firmicutes which employ drastically different DNA replication and repair systems than the widely studied *Escherichia coli*. The materials to produce the *B. subtilis* SSB probe are commercially available, so the methodology described here is widely available unlike previously published methods for the *E. coli* SSB.

## Highlights

• *Bacillus subtilis* specific biosensor has been produced.
• Problem posed by an internal cysteine residue was overcome by site-specific mutagenesis.
• FDA5M is commercially available enhancing the production of *B. subtilis* biosensor..
• *B. subtilis* SSB exhibits salt dependent DNA-binding mode switch like *E. coli* SSB.
• The *B. subtilis* SSB biosensor can be used in cognate reactions and related firmicutes.

## 1 Introduction

The benefit of fluorescent reagentless biosensors to track enzymatic reactions, with minimal disruption to activity has been extensively discussed (Galban et al., 2012). One class of biosensors use protein–fluorophore adducts to create highly specific and sensitive probes that exploit the binding characteristics of the protein component (Gilardi et al., 1994; Salins et al., 2001; Kunzelmann and Webb, 2011) Helicase unwinding is stimulated by proteins that bind to the unwound DNA strands and prevent re-annealing (Dillingham et al., 1999). Wild type (wt) *Escherichia coli* SSB (single-stranded DNA-binding protein) has been used to track helicase activity by monitoring intrinsic tryptophan quenching upon ssDNA (single-stranded DNA) binding (Zhang et al., 2007; Roman and Kowalczykowski, 1989). To enhance the functionality of that probe a SSB-IDCC (N-[2-(iodoacetamido)ethyl]-7-diethylaminocoumarin3-carboxamide) protein adduct was created leading to increased sensitivity and resolution, and the ability to use the probe in complex helicase systems with multiple protein components (Dillingham et al., 2008). Fluorescence assays are used extensively for helicase analysis as the benefits of real-time feedback, high sensitivity and applicability to single molecule or bulk assays outweigh drawbacks, such as fluorescence associated artefacts (Toseland and Webb, 2010). However, the species-specificity of SSB interactions with protein partners limits wide applicability and requires the production of species-specific SSB probes to be used with cognate protein partners. Not all SSBs behave the same when labelled with fluorophores which makes their production challenging. Here, we describe how to produce a Bacillus *subtilis* SSB probe which posed significantly different challenges than published methods for *E. coli* SSB. Our SSB biosensor has significant advantages over other fluorometric helicase tracking systems, such as dsDNA binding dyes which may inhibit the reactions being studied. In addition to the minimal impact of the protein on the reaction, *B. subtilis* SSB binds with high affinity in the nano-molar range, while coumarin and fluorescein-based fluorophores offer excitation and emission wavelength maxima outside that of intrinsic tryptophans.

In the previous work, a variety of fluorophores were attached at two different sites on *E. coli* SSB and the fluorescence increase upon ssDNA binding was screened for each combination (Dillingham et al., 2008). The best combination (SSBG26C-IDCC) gave a 6-fold increase in fluorescence signal. Unfortunately, the IDCC coumarin is not readily commercially available creating a universal applicability problem. SSB coordinates DNA replication and repair processes and acts as a maintenance hub in both *E. coli* and *B. subtilis* (Costes et al., 2010). However, due to crucial differences between DNA metabolism in *E. coli* and *B. subtilis*, cognate SSB probes are required. Tailoring a probe to the system understudy ensures that native interactions lead to unbiased conclusions.

SSB binds, protects and stabilises ssDNA, preventing re-annealing and the formation of ssDNA secondary structures after the helicase has unwound the double strand in DNA replication and repair. SSB also acts as a recruiting scaffold protein and localizes several other proteins essential for various DNA metabolic roles (Toseland and Webb, 2010). In *E. coli* and in *B. subtilis*, SSB has been shown to physically interact with at least 12 proteins facilitating the functional organization of replication forks (Costes et al., 2010), and so the use of cognate SSB proteins as reagentless biosensors is desirable.

This essential function of SSB is well conserved throughout the three domains of life (Glassberg et al., 1979). All SSBs contain N-terminal OB folds (oligosaccharide binding) which specifically bind to ssDNA through electrostatic and base stacking interactions (Raghunathan et al., 2000). These motifs have been observed in other proteins as monomers, dimers, trimers and pentamers (Shamoo et al., 1995; Bernstein et al., 2004; Wold, 1997; Norais et al., 2009). In bacteria the homo tetramer is the most common SSB species and has been best characterized in the case of *E. coli. B. subtilis* also has a homo tetrameric SSB with sequence identity of 39.2% when aligned with the *E. coli* SSB (EMBOSS Water). However, the *Bacillus* SSB has been poorly characterized in comparison with the *E. coli* homologue, for which a wealth of biophysical studies has revealed complex multi-mode interactions with ssDNA (Shereda et al., 2008; Akinyi et al., 2013).

The C-terminal domain of SSB is responsible for interacting with protein binding partners contains a short evolutionary conserved sequence motif (*E. coli* and *B. subtilis* PPMDFDDDIPF and PIDISDDDLPF, respectively). Although the sequences are similar the two organisms do not have identical discreet interactomes, which presents an underlying issue of using non-cognate probes for *in vitro* assays. Additionally, the binding mode switching induced by changing NaCl or Mg^2+^ conditions, which is a key aspect of SSB function in *E. coli* (Lohman and Bujalowski, 1988; Lohman et al., 1988; Kozlov and Lohman, 2011), has not been investigated in the *Bacillus* system. There is also incongruity between these SSBs at a cellular level, as *B. subtilis* has two SSBs working cooperatively (Lindner et al., 2004). The *ssb* gene encodes the essential housekeeping SSB protein involved in DNA replication, whereas the *ssbB* gene encodes the non-essential SSB2, which shares strong sequence homology (63% identity) with SSB in the N-terminal DNA-binding region, but lacks the C-terminal domain. A fundamental mechanistic disparity is that *E. coli* SSB plays a critical role in a three-point switch that mediates RNA primer hand-off from the primase, DnaG, to the polymerase, DnaE, during lagging strand synthesis (Yuzhakov et al., 1999). In contrast, *B. subtilis* SSB is not required for this hand-off. Instead, the RNA primer is passed from DnaG to DnaE *via* a direct physical interaction between the two proteins (Rannou et al., 2013). Such critical functional differences mean it is important to develop a cognate SSB probe that is compatible with the biological system under study. Here, we present a cheap method with commercially available fluorophores to produce a *B. subtilis* SSB biosensor, have characterized its properties and confirmed its use in a gram-positive specific helicase reaction.

## 2 Experimental procedures

### 2.1 SSB probe production

C51V site-specific mutagenesis was carried out using Agilent Technologies QuickChange Site-Directed Mutagenesis Kit on a pET22b template plasmid containing the wt *B. subtilis ssb* gene. For G23C primers 5′-CTTCGTTATACGCCAAACTTCGCGGCTGGT-3′ and 3′-ACCAGCCGCGAAGTTTGGCGTATAACGAAG-5′ were used. Using the same method for C51V, primers 5′-GCCGATTTCATTAATGTTGTCACTGTTAGAAGAC-3′ and 3′-GTCTTCTCCAAGTGACAACATTAATGAAATCGGC-5′ were used. All oligos were purchased from MWG Biotech high-purity salt-free. The protein was expressed, purified and fluorescently labelled as described in Supplementary information.

### 2.2 dT70 Titration

150 μl samples containing 120 nM SSB tetramer were suspended in 25 mM Tris–HCl pH 7.5, 1 mM DTT and 200 mM NaCl. Various dT70 (purchased from MWG Biotech) concentrations ranging from 0 to 200 nM were added to make up the total volume (150 μl). Fluorescence measurements were taken on a Pelkin-Elmer L855 Luminescence Spectrometer. Samples were excited at 493 nm and emission was scanned from 400–600 nm; the highest emission fluorescence was 515 nm. Excitation and emission slit widths were set to 3.0 nm and the scan speed was 150 nm/mm. This setup was used for the titration and plus/minus DNA emission scan from 500 to 600 nm. The mean value of excitation across 515–520 nm was the value plotted in Fig. 3B.

### 2.3 Comparative dT35 and dT70 titrations in low and high salt

Comparative poly-dT titrations were carried out in 200 μl reactions containing 0.125 μM wt SSB tetramer in 10 mM Tris pH 8.1, 0.1 mM EDTA containing either 20 or 200 mM NaCl, as described in Supplementary information. The raw intensities were corrected to account for dilution effects, photobleaching and inner filter effects as described before (Birdsall et al., 1983; Lohman and Mascotti, 1992; Frank et al., 1997). Data were fitted to a tight binding quadratic equation (Morrison, 1969).

### 2.4 Electrophoretic mobility shift assay (EMSA)

SSB-ssDNA binding reactions at comparatively stoichiometric poly-dT to SSB conditions were carried out in 20 mM Tris pH 7.5, 20 mM NaCl and 1 mM DTT. Serial dilutions of the various SSB species (0.125–20 nM) produced the titrations in Fig. 5A and B, and 5 nM of ^32^P labelled dT70 or dT35 (MWG) were present in each 20 μl reaction. Binding reactions were incubated at 37 °C for 20 min before adding native loading dye (DNA loading dye, New England Biolabs) and loading onto 10% w/v acrylamide TBE mini-gels run at 30 min at 180 V. Gels were dried under vacuum and visualized using a molecular imager and associated software (Biorad).

The EMSA under conditions of large excess of SSB (0–50 nM) over dT70 (2.5 pM) shown in Fig. 4 were carried out in a similar manner but in a buffer containing 20 mM Tris pH 7.5, 200 mM NaCl and 1 mM DTT.

### 2.5 SSB-ssDNA association kinetics

Association kinetics were investigated at 25 °C using a stopped flow apparatus (SF-61 SX2, TGK scientific) essentially as described previously (Dillingham et al., 2008) and in Supplementary information. Data shown are the average of four recordings and were fit to single exponentials using GraphPad Prism software to obtain observed rate constants for each dT70 concentration.

### 2.6 AddAB unwinding assay

DNA unwinding experiments were carried out in a stopped flow apparatus (SF-61 SX2, TGK scientific) at 37 °C as described in Supplementary information.

## 3 Results and discussion

### 3.1 Engineering a fluorescent SSB probe

**Fig. 1:**
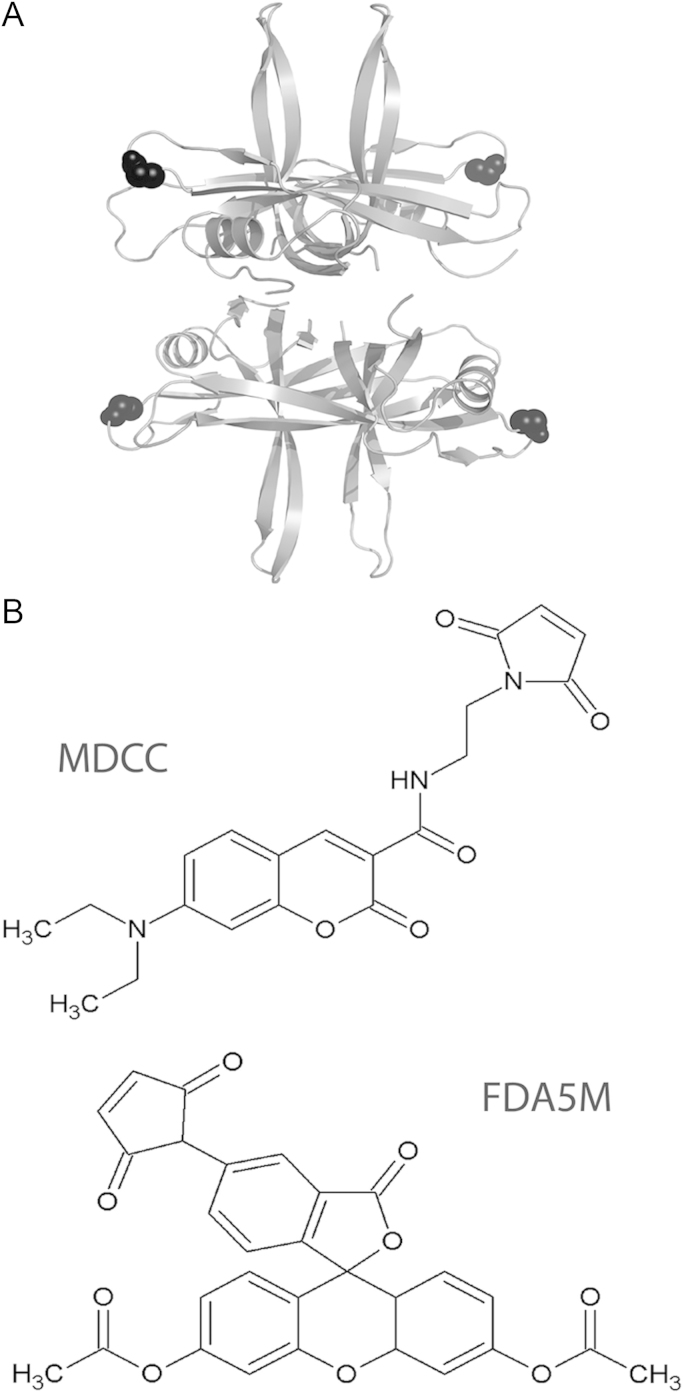
(A) Structural model of the tetrameric *B. subtilis* SSB based upon the structure of *E. coli* SSB (pdb 1EYG, 39.2% identity, 58.6% similarity) with the G23 residue highlighted in black. (B) The structures of the fluorophores, MDCC and FDA5M.

SSB binds ssDNA in a groove that extends around the whole N-terminal domain therefore creating many potentially appropriate fluorophore attachment sites. Previous work in *E. coli* tested multiple fluorophores attached at two sites located on flexible loops on the surface of the protein (S92C and G26C) and a derivative of the G26C mutant gave the largest increase in fluorescence intensity upon binding to dT70 (Dillingham et al., 2008). A ClustalW2 alignment revealed the equivalent residue in *B. subtilis* as G23, and so this site was selected for the attachment (Fig. 1A). To accommodate a maleimide-based attachment to a thiol group, this position was mutated to a cysteine. Wild type (wt) *B. subtilis* SSB already contains another cysteine at position 51 which is predicted to be deeply buried and therefore unlikely to be labelled. Unfortunately, Cys51 was labelled, and so an additional point mutation (C51V) was required to alleviate this issue.

In order to promote universal applicability and transferability of this technique, commercially available fluorophores were selected for testing. Of those tested, fluorescein diacetate 5-maleimide (Fig. 1B) gave significantly less protein loss by precipitation during labelling. Table S1 in Supplementary information presents the percentage of protein loss by precipitation during the labelling reaction described in the Experimental Procedures. The maleimide disrupted the tertiary and quaternary *B. subtilis* SSB structures, possibly by binding to the internal cysteine at position 51. This issue is avoided in the *E. coli* SSB because it does not contain this internal cysteine. The Pfam Hidden Markov Model logo of SSB (PF00436) at this position reveals that after cysteine the most conserved residues are valine and isoleucine, respectively. On this basis we constructed C51V. Table S1 also reveals that FDA5M (fluorescein diacetate 5 maleimide) gives the least protein loss of SSBG23CC51V and was therefore the fluorophore we chose. The maleimide thiol coupling is a highly efficient reaction and mass spectrometry confirmed the efficiency of labelling of this probe, under conditions discussed in the experimental section, to be 100% (Fig. S1 in Supplementary information).

### 3.2 Characterisation of the fluorescent SSB probe

**Fig. 2:**
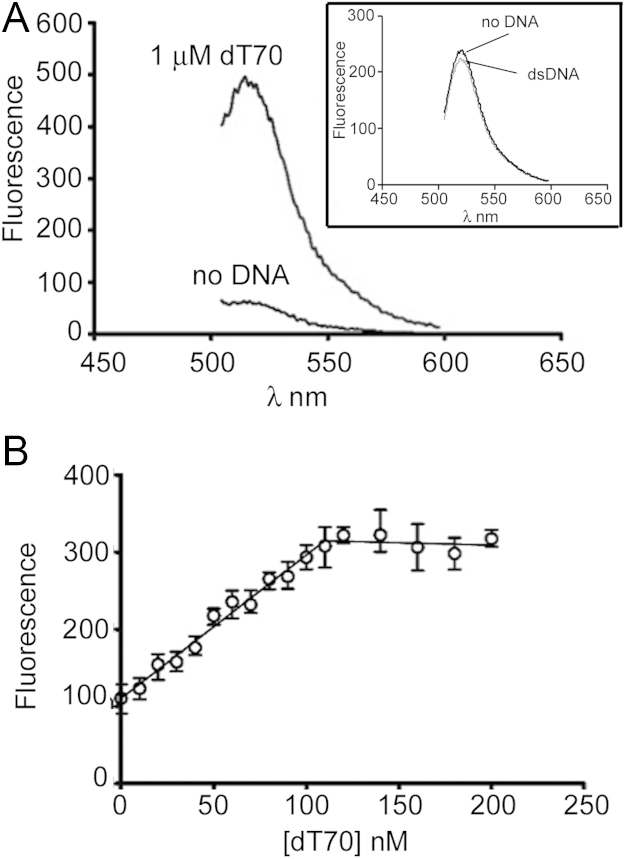
(A) An emission scan of SSBG23CC51V-FDA5M (120 nM) from 500 to 600 nm when excited at 493 nm plus and minus 1 µM dT70, and plus and minus dsDNA (7.4 nM pET28a plasmid equivalent to ~0.57 µM 70mer binding sites inset). (B) Titration of dT70 with SSBG23CC51V-FDA5M (120 nM). The signal increases up to 120 nM dT70 when it reaches a plateau indicating that the SSB is fully bound in a 1:1 stoichiometry with the DNA.

The ability of the SSB-G23CC51V-FDA5M probe to detect ssDNA was investigated using single stranded polythymidylate DNA (dT). Addition of excess dT70 to the probe resulted in a 9-fold increase in fluorescence emission intensity (Fig. 2A), which is 50% higher than the best performing *E. coli* probe. No fluorescence intensity increase was detected upon addition of dsDNA to the probe, showing that the signal is specific to ssDNA as expected (Fig. 2A, inset).

Titration of dT70 into a 120 nM solution of SSB tetramer in Fig. 2B shows a linear fluorescence intensity increase up to a 1:1 stoichiometry, beyond which the signal reaches a plateau. Under these conditions the 70mer is likely to be wrapped around the tetramer engaging with all four subunits as expected by analogy to the well-characterized *E. coli* SSB (Glassberg et al., 1979). No increase in fluorescence could be detected upon addition of excess dsDNA (data not shown). Moreover, this specificity for ssDNA is apparent in helicase assays using the probe (see below).

### 3.3 DNA-binding comparison between wild-type and fluorescent SSBs

**Fig. 3:**
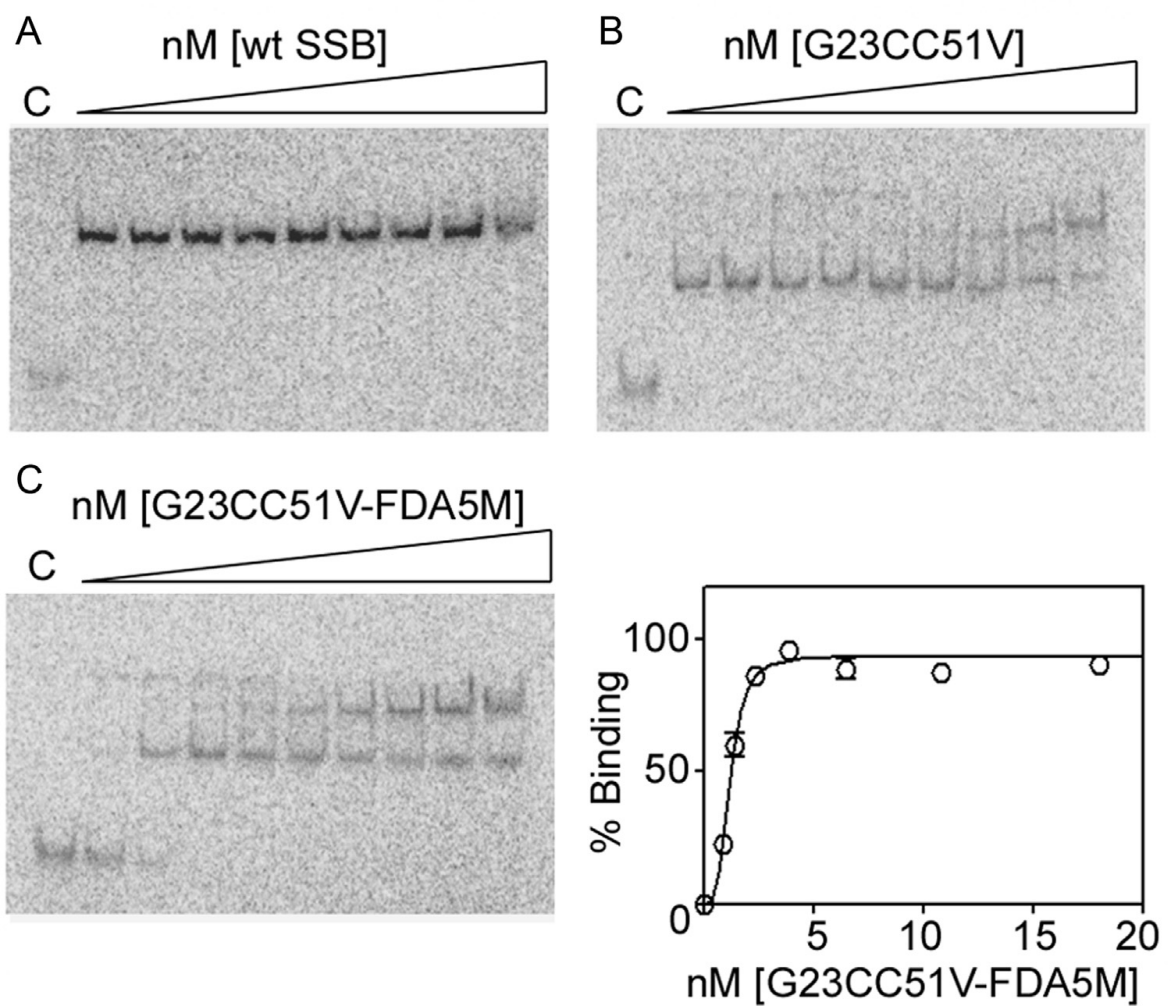
EMSA titrations of wt SSB (A), SSBG23CC51V (B) and SSBG23CC51V-FDA5M (C) in a solution of dT70 (2 .5 pM) at 200 mM NaCl. The concentration range (0.8, 1.4, 2.3, 3.9, 6.5, 10.8, 18 and 50 nM) was the same in all gels and lanes labelled C correspond to control reactions in the absence of SSB proteins. Data from the SSBG23CC51V-FDA5M binding (duplicate gels) were fitted to a one site specific binding with Hill slope equation resulting in *K*_d_ of 1.17 nM.

To ensure the probe is useful for real time assays, both the rate and affinity of ssDNA binding of the mutated and labelled protein must ideally remain high. We characterized binding of wt SSB, SSBG23CC51V and SSBG23CC51V*-*FDA5M to dT70 under conditions of large excess of protein (nM range) over dT70 (2.5 pM) using an electrophoretic mobility shift assay (EMSA) shown in Fig. 3. Binding of wt SSB and SSBG23CC51V to dT70 under these conditions was very tight and did not allow the determination of *K*_d_ values (Fig. 3A and B) but binding of SSBG23CC51V-FDA5M to dT70 was somewhat weaker (Fig. 3C). Fitting the data to an equation of one site specific binding with Hill slope (*R*^2^=0.98) indicated *K*_d_ of 1.17 nM.

**Fig. 4:**
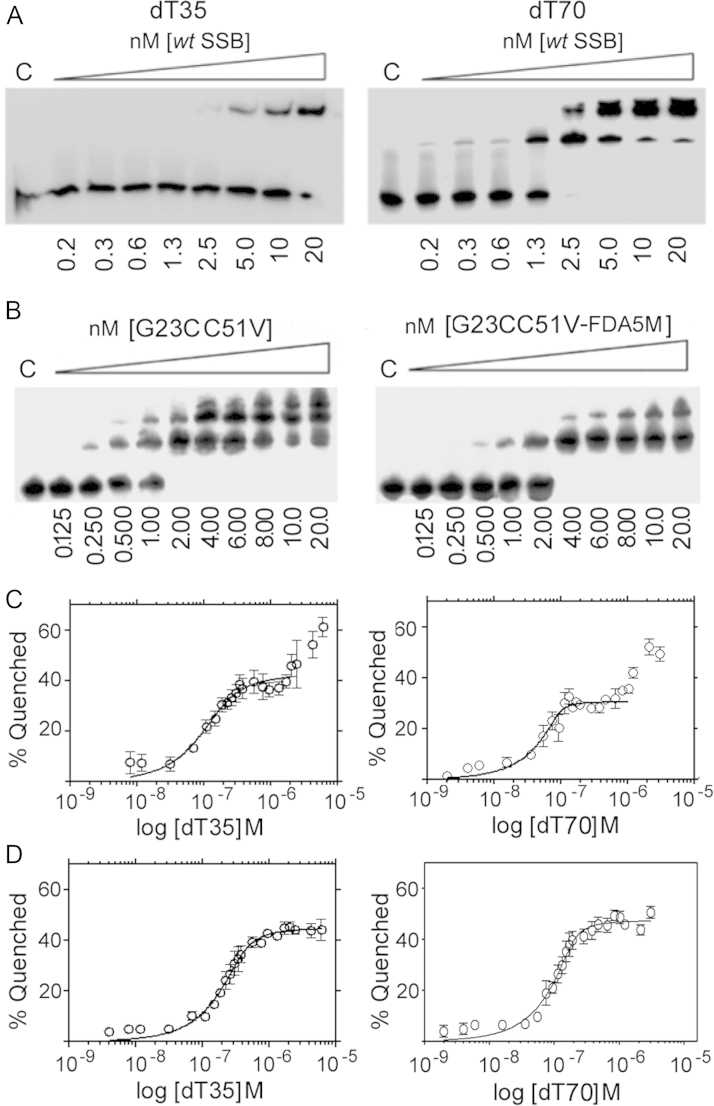
(A) EMSA titrations of wt SSB with dT35 and dT70 at 20 mM NaCl. Lane 1 in each gel labelled C contains no SSB. Binding reactions were carried out in 50 mM Tris pH 7.5, 20 mM NaCl, 5 nM ssDNA (dT35 or dT70) and the SSB (concentrations, as indicated). Samples were incubated at room temperature for 5 min before loading. All mini-gels were 10% w/v acrylamide in TBE and run at 30 min at 180 V. Protein concentrations are shown in nM (referring to SSB tetramer) below each lane in all gels. (B) Representative EMSA gels showing the formation of higher order species when the SSBG23CC51V protein unlabelled (left) and fluorescently labelled (right) binds to dT70. Lane 1 in each gel labelled C contains no SSB. All experiments were carried out with 5 nM dT70 as described in panel (A). (C) Titrations of dT35 and dT70 binding to 0.125 μM wt SSB tetramer shown as tryptophan fluorescence quenching versus concentration of poly-dT in low salt (20 mM NaCl). (D) Titrations of dT35 and dT70 binding to SSB in high salt (200 mM NaCl). dT70 binding to SSB in 200 mM NaCl. All data points have been derived from triplicate experiments and data were fitted to a quadratic equation as described in methods.

Due to its homo-tetrameric nature with its four ssDNA binding sites (OB-fold domains), SSB has the ability to bind DNA in different binding modes dependent on how many domains interact with the DNA (Shereda et al., 2009). Studies of this phenomenon have almost exclusively focused on the *E. coli* SSB in which two major binding modes have been defined. These are called SSB(70) and SSB(35). In the SSB(70) mode all four OB-fold domains are engaged in binding to approximately 65 nucleotides. In SSB(35) only two OB-fold domains are bound to 35 nucleotides. It has been proposed that these modes have a functional *in vivo* role based on alternating inter-tetramer cooperativity. In both modes of binding, SSB can move along ssDNA using a rolling mechanism. This mechanism may allow a second dT70 to interact with an open binding site and eventually displace the original strand. The multiple bands observed with dT70, but not with dT35, in EMSA at stoichiometric conditions as we titrated in higher concentrations of SSB under low salt (20 mM NaCl) conditions are likely to be multiple SSB tetramers engaging with a single strand of dT70 in a 2:1 or 3:1 ratio (Fig. 4A and B). These data also show that SSB binds to dT70 marginally better than to dT35.

Binding of dT35 and dT70 oligonucleotides to *B. subtilis* SSB at low salt (20 mM NaCl) monitored by inherent tryptophan fluorescence quenching revealed the SSB(35) to SSB(70) binding mode switch as observed for the *E. coli* SSB. In *E. coli* SSB this is explained as a reduction in the affinity of ssDNA for the third and fourth OB domains within the SSB tetramer and is particularly pronounced at low salt concentrations, where the SSB(35) binding mode is stabilized. At high salt concentrations, this effect is much less apparent as the SSB(35) binding mode is destabilized and the protein switches to the SSB(70) binding mode without inter-tetramer positive cooperativity (Lohman and Bujalowski, 1988; Lohman and Ferrari, 1994). In *B. subtilis*, SSB binding to both dT35 and dT70 oligonucleotides at 20 mM NaCl compared to 200 mM NaCl reveals a similar switch (Fig. 4C and D). As poly-dT is titrated in a solution of SSB (0.125 μM tetramer) in low salt conditions an initial binding event is apparent followed by a second binding event at higher poly-dT concentrations (Fig. 4C). Fitting the data from the first binding event to a tight binding quadratic equation, as described in the experimental section, produced relatively good fits with *R*^2^ values of 0.902 and 0.863 and *K*_d_ values of 2.2 and 39 nM for dT70 and dT35, respectively. These values are subject to large errors as the experiments were carried out at stoichiometric conditions of ligand (dT70) relative to receptor (SSB). No comparative *K*_d_ values could be obtained for the second binding event as no saturation was reached under our experimental conditions. At high salt conditions the second binding event was not apparent (Fig. 4D) and fitting the data to a tight binding quadratic equation, produced relatively good fits with R^2^ values of 0.9354 and 0.9445 and *K*_d_ values of 9.4 and 36 nM for dT70 and dT35, respectively. The corresponding *L* (ligand; in this case the ligand is the oligonucleotide) concentrations were 0.35 and 0.18 μM consistent with a 2:1 and 1:1 stoichiometry for dT35:SSB_4_ and dT70:SSB_4_, respectively.

Therefore, binding of *B. subtilis* SSB to dT35 and dT70 at low salt concentration suggests that as the concentration of polydT increases the SSB tetramer switches from the SSB(35) to the SSB(70) mode at high polydT concentrations with both dT35 and dT70. At higher salt concentration, *B. subtilis* SSB, like its *E. coli* counterpart, adopts the SSB(70) binding mode with each SSB tetramer binding to two dT35 oligonucleotides and one dT70 oligonucleotide. Collectively, these data show for the first time that, like *E. coli* SSB, *B. subtilis* SSB also exhibits different salt-dependent ssDNA binding modes.

**Fig. 5:**
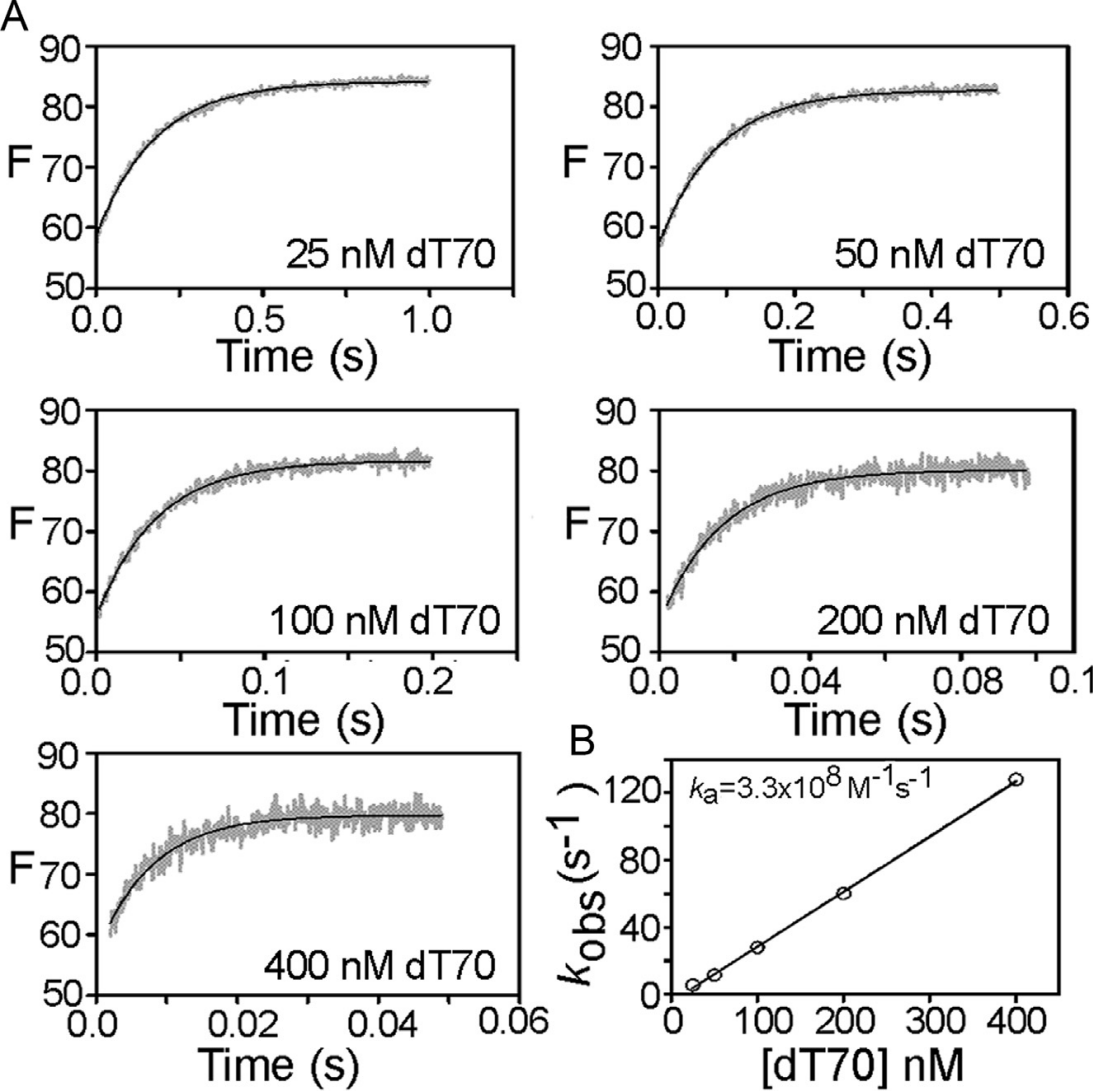
(A) Association kinetics for 5 nM SSB-FDA5M interaction with various dT70 ligand concentrations. The grey traces shown are the average of four recordings and the solid black line is fit to a single exponential to obtain an observed rate constant. (B) Observed rate constants obtained from the individual fluorescence traces from panel A were used to plot the dependence of rate on concentration of DNA. Typically three traces from two separate experiments were averaged to give the data shown.

The bimolecular association rate constant for the interaction of the probe with ssDNA was determined by measuring the binding kinetics under pseudo-first order conditions with a stopped flow apparatus. Free SSB tetramer in solution was rapidly mixed with excess dT70 (at least 5-fold) at varying concentrations and the resulting fluorescence increase monitored with time (Fig. 5). The resulting traces were well-fit to single exponentials which yielded an observed rate constant for each condition. The observed rate constant was proportional to the dT70 concentration up to the highest concentration tested (400 nM), consistent with a simple binding scheme and an association rate constant (*k*_a_) of 3.3×10^8^ M^−1^ s^−1^, which is close to diffusion-limited.

### 3.4 Proof of principle; fluorescent SSB as a functional probe in a cognate helicase assay

**Fig. 6:**
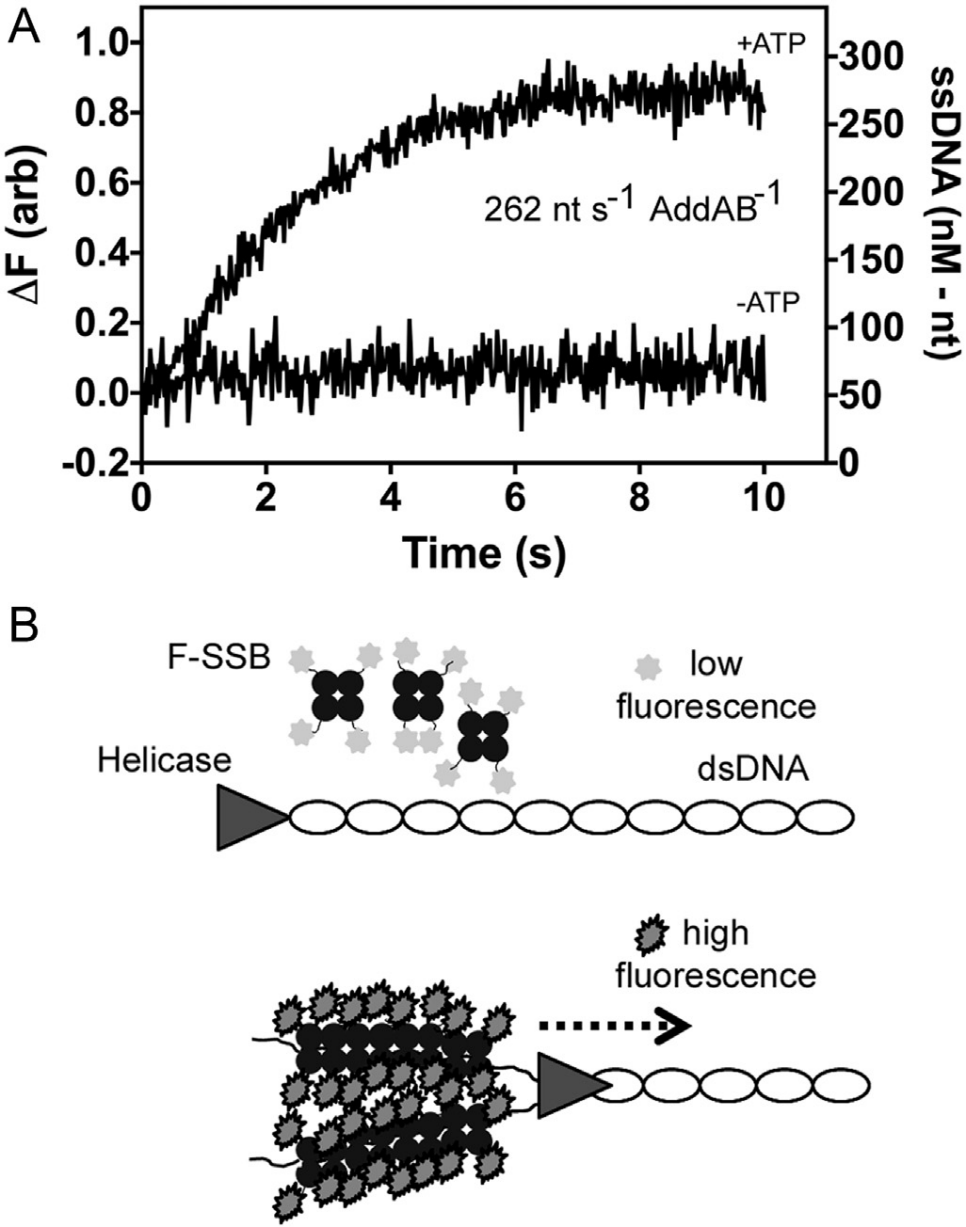
(A) Monitoring the rate of DNA unwinding by AddAB helicase-nuclease using SSBG23CC51V-FDA5M. AddAB-DNA complexes were rapidly mixed with or without ATP in the presence of the SSB probe. In the presence of ATP, the DNA is rapidly unwound at a maximum rate of 262 nM of nucleotides per second per molecule of AddAB. (B) A schematic illustration showing the basis of the SSB biosensor (F-SSB) assay in a helicase reaction. As the helicase translocates forward unwinding the dsDNA, F-SSB binds to the unwound single strands and its fluorescence increases by 9-fold.

The hetero-dimeric AddAB helicase-nuclease is the *B. subtilis* functional homologue of the *E. coli* RecBCD complex and is involved in resecting dsDNA breaks for repair by recombination. AddAB is a powerful helicase, and this activity has been characterized extensively using a variety of different approaches (Saikrishnan et al., 2012; Wigley, 2012). The ability of the probe to detect AddAB-dependent helicase activity was tested using a stopped-flow setup, as previously described (Roman and Kowalczykowski, 1989). Prebound AddAB-DNA complexes were rapidly mixed with ATP in the presence of the probe. Following mixing, there was an ATP dependent increase in fluorescence lasting for several seconds until the reaction completed and the signal reached a plateau (Fig. 6). The signal was calibrated using heat-denatured substrate DNA under the same conditions. This suggested a maximum observed unwinding rate of 52 nM nt s^−1^, equivalent to 260 nt s^−1^ per AddAB enzyme (assuming each DNA end is bound by one AddAB enzyme), which is similar to measurements made under similar conditions using a dye displacement assay (Yeeles et al., 2011).

This experiment provides direct evidence that the *B. subtilis* SSB probe can be used as a helicase unwinding probe *in vitro*. This *B. subtilis* cognate probe provides an alternative to an *E. coli* SSB probe, and is more suitable for systems with closer homology to the gram-positive *B. subtilis* than the gram-negative bacteria. Furthermore, our method refinements act as a template for future work on cognate probe design.

## 4 Conclusion

We have previously demonstrated that *B. subtilis* SSB can be used as a molecular probe to assay PcrA helicase unwinding of an entire plasmid either by Atomic Force Microscopy imaging or real-time utilising intrinsic tryptophan fluorescence changes upon SSB binding to ssDNA (Zhang et al., 2007). However, in complex reactions with multiple proteins inherent fluorescence absorbance by other proteins in the assay interfere with the SSB-mediated signal upon ssDNA binding, making such an assay unreliable. There is a need to develop a cognate SSB biosensor that can be used outside the excitation/emission ranges of tryptophan. Here, we report the construction and characterization of such *B. subtilis* SSB biosensor.

Modifications made to the *B. subtilis* SSB probe allow us to attain high resolution ssDNA binding signals in complex multi-protein assays with high tryptophan backgrounds. Yet despite mutagenesis and chemical modification, the ssDNA specificity and binding efficiency of the SSB biosensor have not been overly perturbed from the wt SSB.

The *B. subtilis* based SSB biosensor gives a 9-fold fluorescence increase upon ssDNA binding, making it suitable as a non-specific real-time unwinding probe as shown using AddAB assays. In addition, unlike previous methods, all materials required for the construction of this probe are commercially available for use with a fully characterized production process detailed above.

Here we present a method to produce a helicase probe and have demonstrated its functionality beyond that already published. Since it is based upon the *B. subtilis* SSB it is particularly suitable for gram positive helicase assays that would benefit from the use of a cognate SSB. In addition we have demonstrated the probes high resolution and functionality in an AddAB helicase assay.
