# Supplementary material for: Engineering a reagentless biosensor for single-stranded DNA to measure real-time helicase activity in Bacillus
Source: Biosens Bioelectron. 2014 Nov 15;61(100):579–86. doi: 10.1016/j.bios.2014.06.011 (PMC4103019; doi:10.1016/j.bios.2014.06.011)
Supplement: Supplementary file 1 — Supplementary material [file mmc1.docx]

**Supplementary Information**

**Engineering a Reagentless Biosensor for Single-Stranded DNA to Mea­sure Real-Time Helicase Activity in** *Bacillus***.**^†^

**Matthew Green***^a^***, Neville S. Gilhooly***^c^***, Shahriar Abedeen*^a^*, David J. Scott***^b^***, Mark S. Dillingham***^c^* **and Panos Soultanas**^*^*^a^*

^a^School of Chemistry, Centre for Biomolecular Sciences, University of Not­tingham, University Park, Nottingham, NG7 2RD, UK

^b^School of Biosciences, University of Nottingham, Sutton Bonington, Leices­tershire, LE12 5RD, UK

^c^School of Biochemistry, Medical Sciences Building, University of Bristol, Bristol BS8 1TD, UK

*Corresponding author; panos.soultanas@nottingham.ac.uk; Tel.: 00441159513525; Fax 00441158468002

**Supplementary Experimental Information**

**SSB Probe Production**

The protein was expressed in Novagen Rosetta(DE3) pLysS cells by growing at 37*^o^*C to an OD (optical density) of 0.6 at 595 nm before induction with 0.5 mM IPTG (isopropyl -D-1-thiogalactopyranoside) and 16 hour induction at 18*^o^*C. Cells were harvested by centrifugation at 6,000g and sonicated at room temperature. Centrifugation at 40,000g at 25*^o^*C ensured the over-expressed SSB remained soluble. A 20% w/v ammonium sulphate cut on the supernatant and subsequent 20,000g centrifugation was done at 4*^o^*C to encourage precipitation. The protein was suspended in 20 mM Tris pH 7.5, 20 mM NaCl and loaded onto a 5 ml HiTrap Q (GE). To elute the protein a gradient from 0-1 M NaCl over 10 column volumes was sufficient (SSB elutes at 35 mS). Gel filtration using a Superdex S75 26/60 (GE) in 20 mM Tris pH 7.5, 200 mM NaCl was then carried out to remove trace contaminants before labelling.

MDCC (N-[2-(maleimidyl)ethyl]-7-diethylaminocoumarin-3-carboxamide) / Fluorescein diacetate 5-maleimide (Sigma) were added in 10-fold excess over monomer to 1 ml of 2 mg/ml SSB. This reaction was left in the dark at room temperature with slow circular turning under nitrogen for 2 hours. The reaction was stopped by adding 3-fold excess DTT (dithiothreitol) to fluorophore. DTT and excess fluorophore were removed using a 5 ml HiTrap desalting column (GE Healthcare) equilibrated in gel filtration buffer followed by 2 hours dialysis in 20 mM Tris pH 7.5, 20 mM NaCl and 20% v/v glycerol in the dark at room temperature for 4 hours.

**Comparative dT35 and dT70 titrations in low and high salt**

Serial dilutions of dT35 or dT70 were prepared in the same buffer with 3 min incubations between measurements. DNA binding was assayed by measuring inherent fluorescence quenching upon DNA-binding. The excitation and emission wavelengths were set at 296 nm and 350 nm with corresponding slit widths at 2 nm and 10 nm, respectively. The temperature of the fluorimeter was carefully maintained at 21*^o^*C and each titration point was averaged over eight measurements.

**SSB-ssDNA association kinetics**

5 nM SSBG23CC51V-FDA5M was rapidly mixed with dT70 at the concentration indicated in a buffer containing 25 mM Tris-Cl pH 7.5, 20 mM NaCl, 1 mM DTT and 5 μM BSA. SSBG23CC51V-FDA5M was excited at 496 nm and the resulting fluorescence above 515 nm was recorded.

**AddAB unwinding assay**

ClaI-linearised pSP73 (0.2 nM) was incubated with the helicase-nuclease AddAB enzyme (25 nM), which was pre­pared as described previously (Yeeles et al., 2011), in a buffer containing BSA (100 μg.ml^-1^ Sigma), Tris-acetate (25 mM, pH 7.5), magne­sium acetate (2 mM), DTT (1 mM) and NaCl (100 mM). This solution was incubated for 2 minutes before mixing against an equal volume of a solution containing ATP (1 mM), BSA (100 μg.ml^-1^, Sigma), Tris-acetate (25 mM, pH 7.5), mag­nesium acetate (2 mM), DTT (1 mM) and NaCl (100 mM). SSBG23CC51V-FDA5M was excited at 496 nm with the slits set at 3 mm and resulting fluorescence above 515 nm recorded. The change in fluorescence associated with 100% unwinding was calibrated by subtraction of the fluorescence value for non-denatured pSP73 (0.2 nM) from denatured pSP73 (0.2 nM). The unwinding rate reported was obtained from the maximum point of the first derivative of the data.

**Supplementary results**

**Table S1** The percentage of protein (SSBG23C and the double mutant SSBG23CC51V) lost after the labelling reaction with MDCC and FDA5M. As labelling efficiency was determined to be 100% by ESI-TOF (see Supplementary Fig. S1), the concentrations of the FDA5M labelled proteins were quantified spectrophotometrically at **λ**495 nm where protein absorbance does not contribute. The molar extinction coefficient of FDA5M is 83,000 M^-1^ cm^-1^. The concentrations of the MDCC-labelled proteins were similarly quantified at **λ**430 nm using the molar extinction coefficient of MDCC 46,800 M^-1^ cm^-1^.

Protein Fluorophore Protein Loss

SSBG23C MDCC 100%

SSBG23C FDA5M 57%

SSBG23CC51V MDCC 50%

SSBG23CC51V FDA5M 12.5%

**Supplementary results**

**
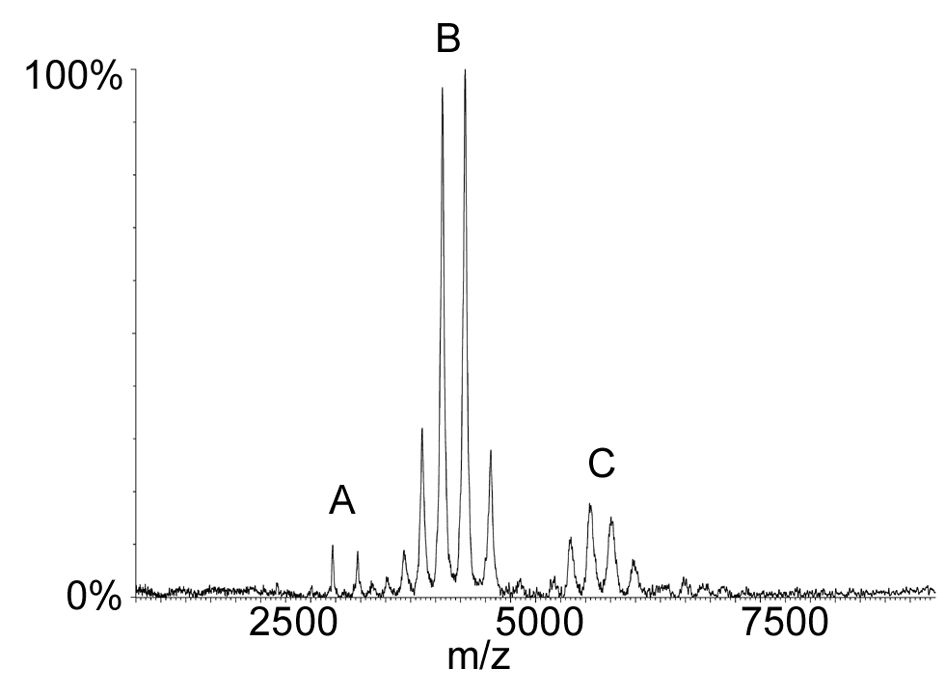
**

**Supplementary Figure S1;** The ESI TOF (Electrospray Ionization Time of Flight) spectra of *B. subtilis* SSBG23CC52V covalently labelled with FDA5M. The data indicated 100% efficiency of labelling based upon the molecular weights (MW) detected. Peaks A, B and C correlate to MW of 38,637.95±19.06, 77,270.65±7.93 and 155,040.00±17.87 Da, respectively. These correspond to theoretical masses of the labels SSB; dimer 38,591.56 Da (-0.12% difference), tetramer 77,183.11 Da (-0.11% difference) and octomer 154,366.22 Da (-0.43% difference) as opposed to unlabelled dimer 37,568.68 Da (2.77% difference), tetramer 75,137.35 Da (2.76% difference) and octomer 150,274.70 Da (3.07% difference), respectively. These differences correlate to the MW of the fluorophore (511.44 Da) which is 2.72% of the overall weight. No lower intensity peaks correlate to the unlabelled protein. Theoretical mass calculated includes N-terminal methionine.
